# EASINESS: *E. coli* Assisted Speedy affINity-maturation Evolution SyStem

**DOI:** 10.3389/fimmu.2021.747267

**Published:** 2021-12-03

**Authors:** Hai-nan Zhang, Jun-biao Xue, Aru Ze-ling Wang, He-wei Jiang, Siva Bhararth Merugu, Da-wei Li, Sheng-ce Tao

**Affiliations:** ^1^ Shanghai Center for Systems Biomedicine, Key Laboratory of Systems Biomedicine (Ministry of Education), Shanghai Jiao Tong University, Shanghai, China; ^2^ Engineering Research Center of Cell and Therapeutic Antibody of Ministry of Education, School of Pharmacy, Shanghai Jiao Tong University, Shanghai, China

**Keywords:** antibody affinity improvement, directed evolution, 18A4Hu, error-prone DNA polymerase I, protein-fragment complementation assay

## Abstract

Antibodies are one of the most important groups of biomolecules for both clinical and basic research and have been developed as potential therapeutics. Affinity is the key feature for biological activity and clinical efficacy of an antibody, especially of therapeutic antibodies, and thus antibody affinity improvement is indispensable and still remains challenging. To address this issue, we developed the *E. coli* Assisted Speed affINity-maturation Evolution SyStem (EASINESS) for continuous directed evolution of Ag–Ab interactions. Two key components of EASINESS include a mutation system modified from error-prone DNA polymerase I (Pol I) that selectively mutates ColE1 plasmids in *E. coli* and a protein–protein interaction selection system from mDHFR split fragments. We designed a GCN4 variant which barely forms a homodimer, and during a single round of evolution, we reversed the homodimer formation activity from the GCN4 variant to verify the feasibility of EASINESS. We then selected a potential therapeutic antibody 18A4Hu and improved the affinity of the antibody (18A4Hu) to its target (ARG2) 12-fold in 7 days while requiring very limited hands-on time. Remarkably, these variants of 18A4Hu revealed a significant improved ability to inhibit melanoma pulmonary metastasis in a mouse model. These results indicate EASINESS could be as an attractive choice for antibody affinity maturation.

## Introduction

As one of top groups of important biological molecules with the ability of specific targeting (1, 2), antibodies have been widely applied in basic research, clinical diagnostics, and therapeutics (3, 4). To date, nearly 79 monoclonal antibody (mAb) drugs have been approved, and over 570 therapeutic antibodies are in the clinical development phase for treating various diseases, including cancer, inflammatory disease, and infectious disease (5–7). Except for mAbs, a wide variety of antibodies have been developed according to different applications and production techniques, including other full antibodies, such as neutralizing antibodies (8, 9), polyclonal antibodies (10), and engineered antibody fragments, such as antigen-binding fragments (Fabs), single-chain variable fragments (scFvs) (11, 12), and VHH single-domain antibodies (13, 14). Due to obvious advantages of small size, strong penetrability, and high specificity, scFvs are widely applied in targeted therapy, intracellular immunity diagnosis, and biological imaging detection (15–17). Generally, antibodies specifically bind to antigens at varied affinities, and the high affinity of an antibody indicates high efficacy of the binding to the antigen, which could probably reduce the antibody dosage and toxicity. High affinity is a key feature for the successful development of therapeutic antibodies. Thus, during development of therapeutic antibodies, antibody affinity improvement is a critical and essential standard procedure.

Current technologies for antibody affinity maturation can be mainly classified into two categories: those based on somatic hypermutation (SHM) and those based on an antibody library. The first strategy exploits the hypermutation characteristics of specific cells, such as B cells, H1299 cancer cells and Raji cells (18, 19). However, this approach strongly depends on specific cells, requires difficult genetic manipulation, and is AID (activation-induced cytidine deaminase)-dependent. The second strategy is also known as directed evolution *in vitro* and usually involves two important components, the construct of an antibody mutation library and a selection system. Approaches for building antibody mutation libraries include error-prone polymerase chain reaction (PCR), site mutagenesis, chain shuffling, DNA shuffling, and complementarity-determining region (CDR) walking (20–23), and the selection systems include phage, yeast, and ribosome display (24–26). Usually, affinity maturation for one antibody requires the construction of an independent library and display selection system. These technologies require building different libraries, which are expensive, time-consuming and labor intensive to produce.

Herein, we developed EASINESS (*E. coli* Assisted Speedy affINity-maturation Evolution SyStem) for fast antibody affinity maturation. EASINESS consisted of two key components, a mutation system modified from error-prone Pol I that selectively mutates ColE1 plasmids in *E. coli* (27) and an antigen–antibody interaction selection system from the mDHFR split fragments (28). The ColE1 plasmid is a low-copy plasmid which can avoid dilution of mutants with high-copy plasmids in *E. coli*. The feasibility of EASINESS was confirmed by reversing mutated GCN4 with three key mutated sites to wild-type GCN4. To test the applicability of EASINESS, we selected an antigen (ARG2)–antibody (18A4Hu) pair, with the potential therapeutics for melanoma (29). In 7 days, with limited hands-on time, we generated 18A4Hu variants with 5~12-fold higher affinity than that of the wild-type 18A4Hu. These variants demonstrated the markedly improved ability to inhibit melanoma pulmonary metastasis in a murine model. Therefore, EASINESS could serve as an option for fast and easy affinity maturation of an antibody of interest.

## Results

### Schematic Diagram and Workflow of EASINESS

**Figure 1:**
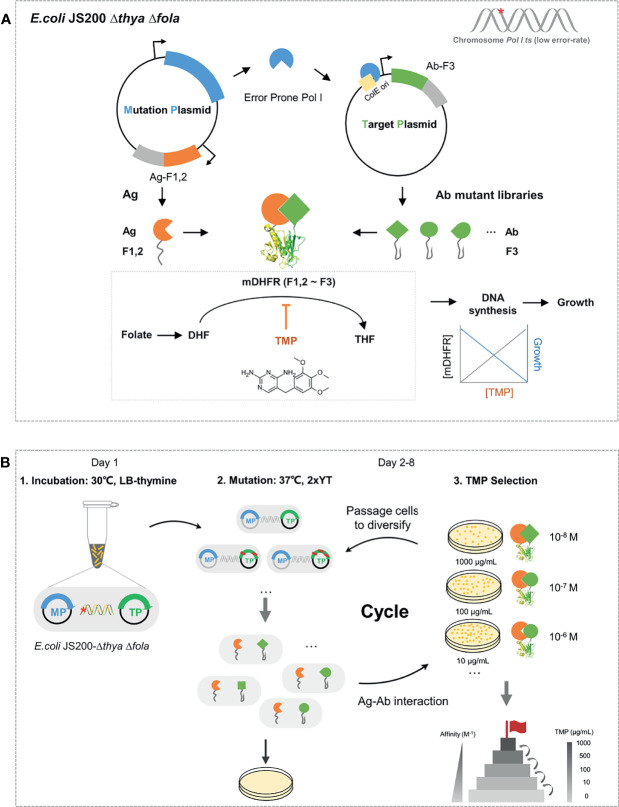
Schematic and workflow of the EASINESS. **(A)** Anatomy of the key cell in EASINESS, i.e., *E. coli* JS200-*ΔthyaΔfola*. *E. coli* JS200-*ΔthyaΔfola* carries two plasmids: the mutation plasmid (MP), which mainly enables mutagenesis, and the target plasmid (TP), which encodes an evolving antibody part (Ab-F3). The antigen part (Ag-F1,2) was expressed in MP. The reconstitution survives on selecting media with trimethoprim (TMP). The resistance to TMP is proportional to the binding affinity between Ag and Ab. The red star indicates *E. coli* JS200 with the gene thya and fola knocked out. **(B)** The workflow of EASINESS. On day 1, the *E. coli* JS200-*ΔthyaΔfola* with MP and TP was grown in LB medium with thymine at 30°C overnight and then was shifted to 37°C in 2× YT medium with thymine to mutagenesis. On days 2–8, mutation and screening at 37°C were conducted. Strains were plated to select positive clones with reconstituted mDHFR activity through Ag-Ab binding. To select Ab with higher affinity to the designated target protein, the positive clones were iterated on plates with higher concentrations of TMP until the concentration reached 1,000 ng/μl. The chemical structural formula of TMP was drawn in InDraw web version (http://www.integle.com/static/indraw).

In EASINESS, error-prone DNA polymerase I, with three key residues mutated, i.e., D424A, I709N, and A759R, is expressed in the pEP mutation plasmid (MP) and hypermutates the gene of interest (GOI) in the target plasmid (TP) with a mutation frequency of 8.1 × 10^-4^ per bp (27). Meanwhile, an affinity maturation selection system is designed based on a protein-fragment complementation assay (PCA) system using murine dihydrofolate reductase (mDHFR), where *E. coli* relies on the interaction between antigen (Ag) and antibody (Ab) to form functional mDHFR for growth under the pressure of trimethoprim (TMP) (**Figure 1A**). TMP, as an antifolate antibiotic, is an inhibitor of dihydrofolate reductase (DHFR) and bacteriostatic by blocking up the bacterial DNA synthesis process and folate metabolism, having a 12,000-fold lower affinity of binding to human or murine DHFR than bacterial DHFR (28). Error-prone Pol I in *E. coli* can preferentially mutate GOI in TP with ColE1 ori at a mutation rate of 10^-3^ substitutions per base and a much lower probability of mutagenesis in chromosomes (27). To avoid possible leakage caused by endogenous dihydrofolate reductase, we knocked out endogenous genes *folA* and *thyA* (30), which respectively encode dihydrofolate reductase and thymidylate synthase in *E. coli* (**Figure S1A**). When Ag that binds to Ab, F1,2 is brought close to F3 to form the whole functional mDHFR (F1,2~F3), the activity of murine dihydrofolate reductase is proportional to the level of TMP (31). EASINESS enables the rapid evolution of antibody affinity improvement through several rounds of mutagenesis and selection (**Figure 1B**). In short, EASINESS works as follows: when antibody fragments are encoded on TP, the *E. coli* strain that we constructed will diversify antibodies through mutation, and after several rounds of growth selection with TMP for antigen binding, clones of high-affinity antibodies are rapidly generated. Thus, only very limited hands-on time is required for performing EASINESS.

### EASINESS Promotes Mutagenesis and Selection of Protein–Protein Interactions

To determine the feasibility of mutagenesis in *E. coli* JS200-*ΔthyaΔfola* cells, we performed *β*-lactamase reversion assay. On the medium plates with 50 μg/ml carbenicillin, very few clones were obtained when wild-type Pol I (pWT) was tested, while many clones were obtained when error-prone Pol I (pEP) was tested. We randomly selected five clones from each of these two plates; 100% of clones were reversely mutated for pEP, while few were mutated for pWT (**Figure S1B**). Next, we examined mDHFR-based PCA and found that cells carrying the pair of GCN4-F1,2 and GCN4-F3, as the positive control, grew well when 10 μg/ml TMP was added (**Figure S1C**). These results clearly show that mutagenesis and selection of protein–protein interactions work well in the *E. coli* JS200-*ΔthyaΔfola* strain.

### Reversing Site Mutation of GCN4^3mut^ Using EASINESS

**Figure 2:**
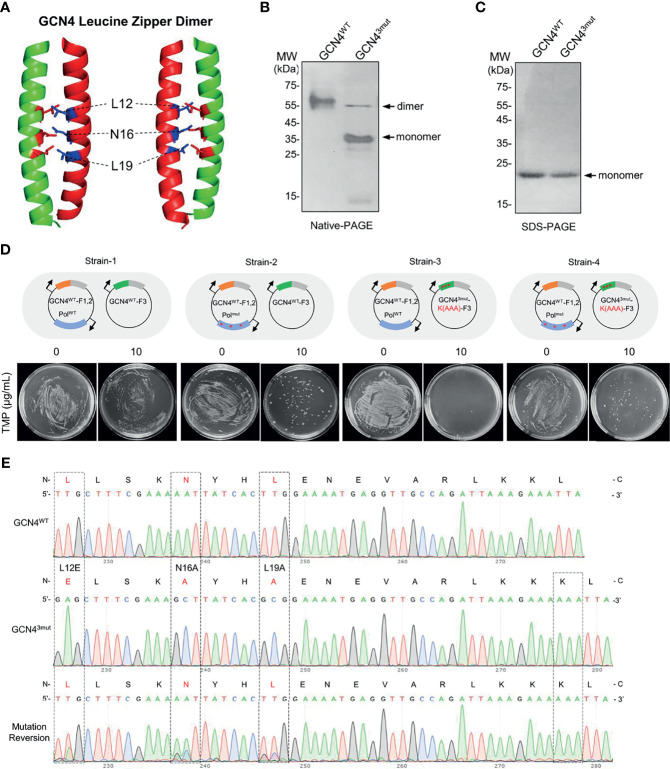
Recovery of the homodimer formation activity in mutated GCN4 by EASINESS. **(A)** The structure of the GCN4 leucine zipper dimer (PDB: 1CE9). **(B, C)** Native and SDS-polyacrylamide gel electrophoresis (PAGE) analysis of the dimer formation of GCN4^WT^ and GCN4^3mut^ (L12E, L19A, and N16A). **(D, E)** Reversing the mutation of GCN4^3mut^ using EASINESS. Strain-4 carrying the mutation plasmid pEP (PolI^3mut^) was grown in the presence of 10 μg/ml TMP, and three mutation sites were reversed from GCN4^3mut^ to GCN4^WT^ according to Sanger sequencing. Strain-1: GCN4^WT^-F1,2 and GCN4^WT^-F3 with pWT (PolI^WT^), Strain-2: GCN4^WT^-F1,2 and GCN4^WT^-F3 with pEP (PolI^3mut^), Strain-3: GCN4^WT^-F1,2 and GCN4^3mut^-F3 with pWT, Strain-4: GCN4^WT^-F1,2 and GCN4^3mut^-F3 with pEP. An additional mutation (AAA) at the end of GCN4^3mut^ was applied as a marker to differentiate the reversed mutant from wild-type GCN4.

To determine whether EASINESS could improve the binding affinity of interacting proteins, we selected the “leucine-zipper” dimerization element of GCN4 as the model. GCN4 performs its transcriptional regulation of amino acid biosynthesis in yeast (32) through a leucine zipper homodimer at the C-terminal end of GCN4 binding to DNA with a known dimerization affinity of 1.8 × 10^-10^ M (33). By the structural analysis of the GCN4 leucine zipper dimer, we identified three key sites (L12, N16, L19) for GCN4 dimer formation (data not shown). We mutated these three sites as L12E, N16A, and L19A and named this construct GCN4^3mut^ (**Figure 2A**). Gel electrophoresis showed that under nondenaturing conditions, the majority of GCN4^3mut^ was monomeric, while almost 100% GCN4^WT^ formed dimers (**Figures 2B, C**). These results indicate that the strong interaction between the GCN4 leucine zipper homodimer is significantly disrupted by introducing L12E, N16A, and L19A.

To test whether the weak binding affinity of GCN4^3mut^ could be enhanced by EASINESS, we constructed four plasmids, i.e., pWT-GCN4-F1,2, pEP-GCN4-F1,2, pLA230-GCN4^WT^-F3, and pLA230-GCN4^3mut^-F3. As demonstrated in **Figure 2D**, four combinations of these plasmids were designed and co-transformed into *E. coli* JS200-*ΔthyaΔfola* cells, designated Strain-1, Strain-2, Strain-3, and Strain-4. In the absence of selection pressure (without TMP), all four strains grew normally. When 10 μg/ml TMP was applied, as expected, there was no growth for Strain-3 since WT Pol I was included on MP, while obvious growth was observed for Strain-4 with error-prone Pol I (pEP) (**Figure 2D**). Surprisingly, after only one round of selection, all three sites were mutated back to original residues with a theoretically low possibility. Considered that a possible cross contamination with GCN4^WT^ during growth, we intentionally added three nucleotides (AAA) to the C-terminus of GCN4^3mut^ as a marker. Sanger sequencing confirmed the complete reversion of GCN4^3mut^ by EASINESS (**Figure 2E**). These results demonstrate the capability of EASINESS to improve the binding affinity of two interacting proteins.

### Binding Affinity Improvement of 18A4Hu^scFv^ to Its Target AGR2

Anterior gradient 2 (AGR2) encodes an endoplasmic reticulum (ER)-resident protein that belongs to the protein disulfide isomerase family and is usually secreted into the extracellular matrix (ECM) and plays a pivotal role in tumor microenvironment development (34). The expression of AGR2 is elevated in multiple cancers, including lung cancer, breast cancer, prostate cancer, and ovarian cancer (35). AGR2 is detectable in many cancer patients and thus could serve as a potential biomarker for cancer diagnostics (36). Humanized 18A4 (18A4Hu) and murine 18A4 antibodies specifically bind to AGR2 and exhibit some tumor growth inhibition activity (29, 37, 38).

**Figure 3:**
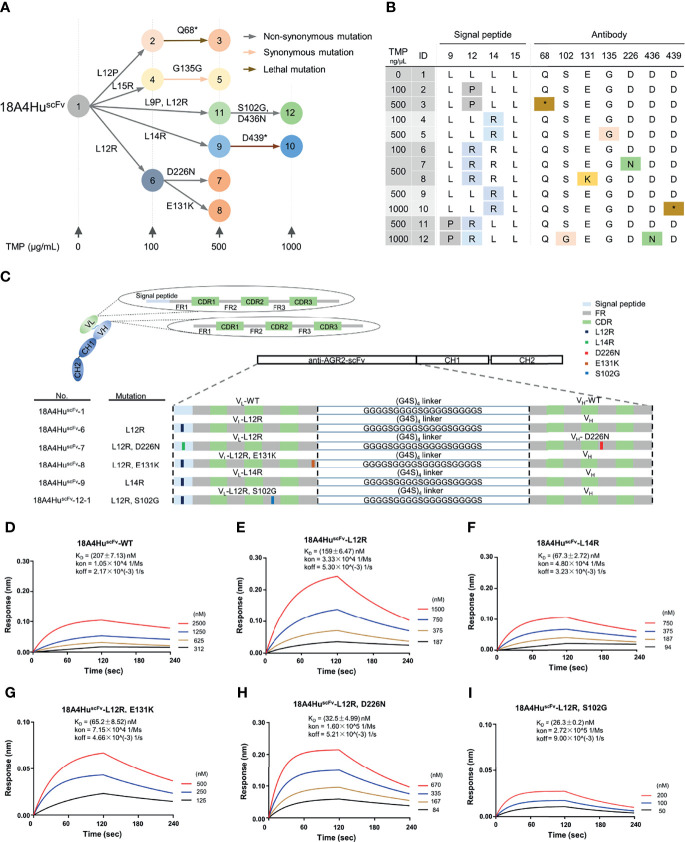
Improved binding affinity of 18A4Hu^scFv^ to its target protein by EASINESS. **(A)** Plausible evolution trajectories of 18A4Hu^scFv^ by EASINESS in gradient concentrations of TMP from 0 to 1,000 (μg/ml). **(B)** These mutated sites of 18A4Hu^scFv^ located in the signal peptide or antibody backbone. The red star indicates stop codon. **(C)** The location of mutated residues from five variants in different domains of 18A4Hu^scFv^. **(D–I)** Biolayer interferometry (BLI) assays for measuring the binding affinity to AGR2 of select 18A4Hu^scFv^ mutants.

To test whether EASINESS could generate high-affinity 18A4Hu antibodies for ARG2 in *E. coli* over a short period of time, we sought to improve the binding affinity of 18A4Hu starting from scFv. Based on a well-established model, we performed affinity improvement of 18A4Hu^scFv^ through repetitive mutagenesis and selection in cycles with gradient concentrations (0, 100, 500, 1,000 ng/μl) of TMP in *E. coli* (**Figure 1B**). By means of colony PCR and Sanger sequencing, we finally obtained 12 variants with single and cumulative mutations and depicted a plausible evolutionary trajectory of 18A4Hu^scFv^ on a small scale (**Figure 3A**). On the basis of evolutionary trajectory analysis of these mutations, we chose five variants (6, 7, 8, 9, 12-1) distributed in three different selection pressures and in two branches of the evolutionary path (**Figures 3A, B**). Mutations of selected variants (L12R, L14R, S102G, E131K, and D226N) were randomly distributed on 18A4Hu^scFv^ domains containing signal peptides, VL and VH (**Figures 3C**, **S2A**). 18A4Hu^scFv^ variants encoding EASINESS-derived consensus mutations exhibited different extents of higher AGR2-binding affinities, measured by biolayer interferometry (BLI), compared to 18A4Hu^scFv^ wild-type (**Figures 3D–I**). Specifically, 18A4Hu^scFv^-6 selected under 100 ng/μl TMP exhibited a two-fold affinity improvement, while 18A4Hu^scFv^-7, 18A4Hu^scFv^-8, and 18A4Hu^scFv^-9 selected under 500 ng/μl TMP exhibited 5~7-fold affinity improvements, and 18A4Hu^scFv^-12-1 selected under 1,000 ng/μl TMP exhibited a 12-fold affinity improvement. These results showed a strong correlation between the TMP concentration and antibody binding affinity (**Figure S2B**).

It is worth noting that an N-terminal signal peptide that facilitates the expression of scFv to periplasm was included. We adopted this signal peptide (SP) from a 18A4Hu scFv construct (39). The SP is originally from the variable region of the human IGKV1-27 immunoglobulin light chain (GenBank: MN197480.1, from nucleotides 1 to 66). It is no doubt that this peptide functions (secrets to periplasm and then be cleaved) in human cells; however, it is not clear whether this is also the case when the SP is expressed in *E. coli*. To test this, we have examined the periplasmic and cytoplasmic expression of SP-18A4Hu^scFv^-WT, SP-18A4Hu^scFv^-L12R, and SP-18A4Hu^scFv^-L14R; 18A4Hu^scFv^-WT (with SP removed) was included as control. In addition, a construct with prokaryotic signal peptide DsbA1 (40) (DsbA1-18A4Hu^scFv^-WT) was also added as a positive control. The results (**Figure S4**) clearly showed that the SP hardly works in *E. coli*, and >99.9% of the 18A4Hu-scFvs were in cytoplasm without the cleavage of the SP.

### 18A4Hu Variants Enhance the Inhibition of Tumor Metastasis in the Lungs of BALB/c Nude Mice

**Figure 4:**
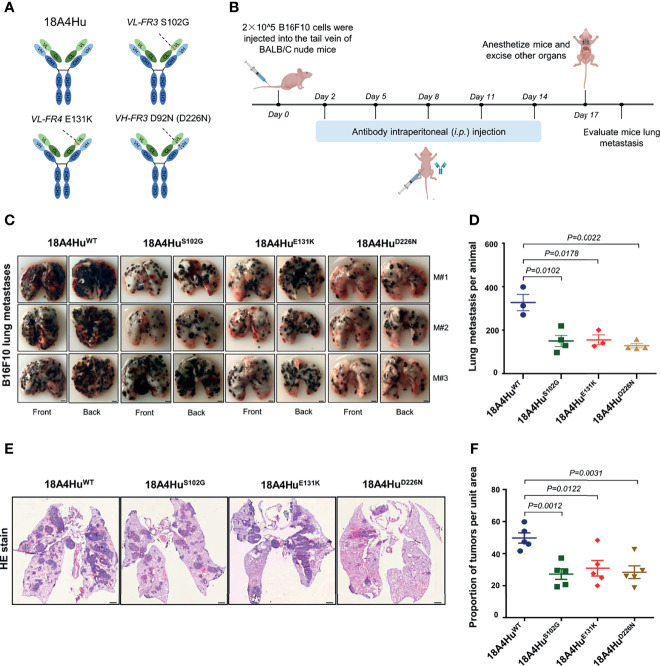
18A4Hu variants inhibit tumor metastasis in the lungs of BALB/c nude mice. **(A)** Three scFv mutants (S102G, E131K, or D226N) were placed back to the human IgG backbone. **(B)** Schematic illustration of the treatment design. BALB/c nude mice were implanted with 5 × 10^5^ per mouse B16F10 tumor cells. Day 2 after injection, mice were treated with the antibody 18A4Hu and its variants by intraperitoneal (i.p.) injection (100 mg per kg in 200 μl 1 × PBS) one for every three days for five times. **(C, D)** Representative macroscopic images **(C)** of lungs from 18A4Hu- and variant-treated animals to quantify lung metastases **(D)** after *i.v.* engraftment of B16F10 cells. **(E, F)** Representative macroscopic images of H&E staining in lung **(E)** and quantification of metastatic pulmonary metastasis nodules **(F)** from 18A4Hu- and variant-treated animals. Data are represented as the mean ± s.e.m. of n = 3/4 mice **(D)**, n = 5 **(F)**. *p* values calculated with unpaired Student’s t-test **(D, F)**. Scale bar, 2 mm **(F)**, or 1 mm **(F)**.

To demonstrate the therapeutic potential of antibodies, the best option is to test the full-length antibody physiologically since predominant antibody drugs are in full-length IgG format and contain an available Fc region. To convert the scFv format into a full-length IgG, we respectively subcloned the three mutations, i.e., S102G, E131K, and D226N, from 18A4Hu^scFv^ into full-length 18A4Hu (4), and the 18A4Hu wild type was set as the control (**Figure 4A**). These antibodies were then expressed in HEK293F cells and affinity purified (**Figure S3A**).

To test whether 18A4Hu variants could exhibit effectiveness *in vivo*, we utilized three variants 18A4Hu^S102G^, 18A4Hu^E131K^, and 18A4Hu^D226N^ to treat BALB/c nude mice injected with B16F10 cells and evaluated the inhibition of melanoma pulmonary metastasis in mice (**Figure 4B**) and the body weight of the mice (**Figure S3B**). We observed that the lung metastasis nodules were significantly reduced by 18A4Hu^S102G^, 18A4Hu^E131K^, and 18A4Hu^D226N^ at the end of treatment (**Figures 4C, D**) in comparison to that of 18A4Hu^WT^ treatment, while there was no significant change in body weight during the antibody injection period (**Figure S3B**). Tumor tissues in the lung were harvested for further analysis. Similarly, hematoxylin and eosin (H&E) staining in lung tissue showed a notable decrease in metastatic nodules for three variants, compared with that of 18A4Hu^WT^ (**Figures 4E, F**). Together, these results indicate that these three mutations of 18A4Hu, i.e., S102G, E131K, and D226N, enhance the inhibition of tumor metastasis *in vivo*.

## Discussion and Conclusion

The aim of this study is to develop a highly efficient and easy-to-use strategy to improve the affinity of antibodies of interest. We have developed EASINESS by incorporating error-prone Pol I and the mDHFR split and complementary system in *E. coli*. The feasibility of EASINESS has been validated by reversing the homodimer formation capability of GCN4 from a mutant with three key mutated sites. We have improved the binding affinity of monoclonal antibody 18A4Hu to its target ARG2 12-fold in a few days, proving the applicability of EASINESS for mAbs affinity improvement. The applicability of 18A4Hu mutants has been further confirmed by significantly improving the ability of inhibition melanoma pulmonary metastasis in a mouse model.

There are several advantages of EASINESS. First, EASINESS is a novel *E. coli*-assisted continuous directed evolution strategy that could be directly applied for antibody affinity maturation. Two key steps, i.e., mutagenesis and screening, are seamlessly incorporated in *E. coli*. Except for minimum plating, no additional operation or sophisticated device is required. EASINESS is generally applicable, while other continuous directed evolution systems, such as PACE (41) and OrthoRep (42, 43), are theoretically applicable for this purpose but may lack generality. In addition, compared with a novel strategy of AHEAD based on the OrthoRep mutagenesis and yeast surface display screening (44), EASINESS still has the advantage of simplicity and operability. Second, in comparison to conventional methods of antibody affinity maturation, such as phage display-based screening (24), EASINESS does not require library cloning or other discrete, time-intensive steps but is rather a fast, easy, and maneuverable method.

There are also some limitations of EASINESS. First, more mutations will accumulate in the *E. coli* genome. Although error-prone Pol I is specific for ColE1 origin, there is still leakage (27). The accumulation of mutations in the *E. coli* genome raises the risk of growth inhibition or death and defects in the machinery necessary for selection, i.e., the split and complementary system of mDHFR, even when endogenous dihydrofolate reductase is completely removed by *folA* knockout. One possible way to reduce mutagenesis in the *E. coli* genome is to combine EASINESS with CRISPR-Cas9, which can enhance the mutation specificity through error-prone DNAP fused to dCas9 (45). Second, gene circuits of EASINESS in its current form are not tightly controlled, especially for the control of protein expression. Thus, the level of the complemented and recovered mDHFR, as the index of selection results not only from the enhancement of antibody binding to its target but also from the higher expression level of Ab-F3 during mutagenesis, suggesting that some mutagenesis is irrelevant to the antibody affinity. Possible solutions to this problem include protein codon optimization and building and combining of a series of elements (i.e., different promoters, RBS) as toolboxes to enhance the selection stringency (46) and success rate. Third, the overall better performance of the mutated antibodies may at least be partially due to the increase in the stability, not just higher affinity. Since the final real-world application is the key, we welcome the improvement for both stability and affinity of the target antibody. However, the underlying mechanism of stability improvement may worth further investigation. Finally, for EASINESS, due to the intrinsic characteristics of error-prone Pol I, mutations were well distributed across the first 650 bp (27). Thus, although EASINESS works well with small proteins, such as scFv proteins, in this study, it may not be suitable for large proteins.

In the future, EASINESS could be readily integrated with existing antibody libraries, such as nanobodies or scFvs, thus enabling a “2 in 1” strategy for direct antibody screening and affinity maturation. Clearly, in addition to high-affinity single-chain antibodies, EASINESS may also be used for affinity improvement of ordinary PPIs and is especially suitable for protein-peptide interactions (**Figure 2**). Meanwhile, in some cases, EASINESS could increase protein solubility (data not shown), which is another interesting direction of application. Additionally, if EASINESS is combined with the microbial evolution and growth arena (MEGA) plate (47), the manual transfer of strains from low to high [TMP] could be replaced by microbial self-migration. This approach would greatly simplify the operation procedure of EASINESS and finally enable self-continual directed evolution, almost without any manual interruption. In addition, EASINESS for antibody affinity maturation not only will contribute to the development and quality improvement of antibody drugs but also may provide a better understanding of the function of the target antigen through antibody-antigen interactions.

Taken together, EASINESS is a novel directed evolution system to enable efficient, easy, and fast antibody affinity maturation. In its current form, it is suitable for small-size antibodies, such as scFvs. We believe that EASINESS will shed light on the development of high-affinity antibodies for both basic research and therapeutics.

## Materials and Methods

### General Methods and Cloning

Plasmids used in this study are listed in **Supplementary Table 1**. Oligonucleotides are listed in **Supplementary Table 2**. PCR were performed using KOD FX or KOD plus neo (TOYOBO, Osaka, Japan). Lac-promoter-GCN4-F1,2 and lac-promoter-AGR2-F1,2 constructs were cloned into Bsu36I-digested pWT/pEP PolI backbone (27) with chloramphenicol resistance. GCN4^WT^-F3 and lac-promoter-18A4Hu^scFv^-F3 constructs were cloned into SacI/AflIII-digested pLA230 backbone (27) with kanamycin resistance. Plasmids were constructed using the ClonExpress ^®^ II One Step Cloning Kit (Vazyme Biotech, Nanjing, China), and those with site-directed mutagenesis were generated by PCR with a primer pair for site mutation using KOD Plus Neo.

### Construction of Mutation Vectors and Expression Vectors

Based on pLA230-GCN4^WT^, the reporter plasmid pLA230-GCN4^3mut^ with L12E, N16A, and L19A was generated *via* PCR with KOD Plus Neo (TOYOBO, Osaka, Japan) and primer pair for site mutation. Following digestion of GCN4^WT^ with DpnI (NEB, Ipswich, MA), the resulting plasmids were transformed into DH5α. The pLA230-GCN4^3mut^ were sequenced correctly and then transformed into JS200-*ΔthyaΔfola* cells for mutant reversion screening. Similarly, the expression vectors of pET28a-GCN4^3mut^ and pET28a-18A4Hu^scFv^ mutants were constructed for protein purification.

### JS200-ΔthyaΔfola Strains

*E. coli* JS200-*ΔthyaΔfola* cells were constructed with both *thyA* and *folA* knockout based on JS200 cells using Lambda-Red homologous recombination technology (27, 30, 48), the resulting complete genotypes of which are *SC-18 recA718 polA12 uvrA155 trpE65 lon-11 sulA1 thyA folA*. Cells were grown in Luria–Bertani (LB) medium supplemented with 50 μg/ml thymine or in LB medium supplemented with *thyA* and *folA* genes at 30°C with polA12 (*ts*) knockdown at 37°C.

### β-Lactamase Reversion Assay

*β-*lactamase reversion assay was performed as previously described (27)*. E. coli* JS200-*ΔthyaΔfola* cells were co-transformed with the Pol I plasmid (cm^r^) pWT/pEP and the reporter plasmid (kan^r^) pLA230. Transformed colonies were grown in LB medium with 50 μg/ml thymine, 50 μg/ml kanamycin, and 30 μg/ml chloramphenicol at 30°C to an OD_600_ of 0.4–0.6. For mutagenesis, cultures were diluted 1:200 in 2× YT media with 1 mM isopropyl-β-d-thiogalactoside (IPTG) and grown for 15–17 h in a 37°C shaker. Reversion of the β-lactamase ochre codon was detected by plating the appropriate dilution of the saturated cultures onto kanamycin and chloramphenicol plates containing 50 μg/ml carbenicillin.

### mDHFR-Fragment Complementation Assay

mDHFR-fragment complementation assay was performed as previously described (31)*. E. coli* JS200-*ΔthyaΔfola* cells co-transformed with pWT/pEP-GCN4^WT^-F1,2 and pLA230-GCN4^WT^-F3, pWT/pEP-PknB-F1,2 and pLA230-GCN4-F3, and pWT/pEP-F1,2 and pLA230-F3 only were included as controls. Transformed colonies were grown in LB medium with 50 μg/ml thymine, 50 μg/ml kanamycin, and 30 μg/ml chloramphenicol at 30°C overnight. Cultures were washed with LB medium without thymine and then plated on kanamycin and chloramphenicol plates with 50 μg/ml thymine or 10 μg/ml trimethoprim (TMP) at 30°C.

### GCN4^3mut^ Mutational Reversion Assay

*E. coli* JS200-*ΔthyaΔfola* cells were co-transformed with the pWT/pEP-GCN4^WT^ plasmid and the reporter plasmid (kan^r^) pLA230-GCN4^WT/3mut^, meanwhile being supplemented with the thya gene, to produce Strain-1 (pWT-GCN4^WT^ and pLA230-GCN4^WT^), Strain-2 (pEP-GCN4^WT^ and pLA230-GCN4^WT^), Strain-3 (pWT-GCN4^WT^ and pLA230-GCN^3mut^), and Strain-4 (pEP-GCN4^WT^ and pLA230-GCN4^3mut^). Transformed colonies were grown in LB medium with 50 μg/ml thymine, 50 μg/ml kanamycin, 30 μg/ml chloramphenicol, and 100 μg/ml ampicillin at 30°C until OD_600_ reached 0.4–0.6. For mutagenesis, cultures were diluted 1:200 in 2× YT media supplemented with IPTG and grown for 15–17 h in a 37°C shaker. After being washed with thymine free LB medium, the four strains with its corresponding accessory were plated onto plates containing 50 μg/ml thymine and TMP-free media, or thymine-free and 10 μg/ml TMP media at the appropriate dilution. Reversion of GCN4^3mut^ was detected by amplification using primer pairs on the pLA230 and Sanger sequencing.

### Screening of 18A4Hu Variants by EASINESS

*E. coli* JS200-*ΔthyaΔfola* cells were co-transformed with the modified mutagenesis plasmid pEP-AGR2-F1,2 and target plasmid pLA230-18A4Hu^scFv^-F3. Colonies were grown in LB medium with 50 μg/ml thymine, 50 μg/ml kanamycin, and 30 μg/ml chloramphenicol at 30°C overnight and transferred into thymine-free 2× YT supplemented with 1 mM IPTG to induce mutagenesis. Cultures were washed with thymine-free LB medium and grown on LB agar with 0 μg/ml TMP at 30°C overnight. Five colonies were picked randomly and dissolved in 2× YT liquid medium. One aliquot was subjected to colony PCR using pLA230 primers and Sanger sequencing to assess mutated sites. Another aliquot was used for the next round of mutagenesis with a higher concentration (100, 500, 1,000 μg/ml) of TMP until the concentration reached 1,000 μg/ml.

### Protein Preparation

C-terminal 6× His-tagged GCN4, GCN4^3mut^, and AGR2 and all scFv mutants were cloned into pET28a. Proteins were expressed in BL21 by growing recombinant BL21 cells in 500 ml LB medium to an OD_600_ of 0.4–0.6 at 37°C. Protein expression was induced by the addition of 1 mM IPTG before incubating cells overnight at 16°C. Proteins were purified on Ni-NTA affinity beads and stored at -80°C. For the extraction of periplasmic proteins, SP-18A4Hu^scFv^-WT, SP-18A4Hu^scFv^-L12R, SP-18A4Hu^scFv^-L14R, 18A4Hu^scFv^-WT (without SP), and DsbA1-18A4Hu^scFv^-WT were cloned into pET28a. Proteins were expressed in BL21 by growing recombinant BL21 cells in 50 ml LB medium to an OD_600_ of 0.4-0.6 at 37°C. Proteins were induced by the addition of 1 mM IPTG before incubating cells overnight at 16°C. The periplasmic proteins were extracted according to a previously described method (49).

### Affinity Measurement by Biolayer Interferometry Assay (BLI)

Biolayer interferometry assays were performed on an Octet Red 96 (ForteBio) instrument at 25°C with shaking at 1,000 rpm. Streptavidin biosensors were incubated in SD buffer (1× PBS, 0.02% Tween-20, 0.1% BSA) for 20 min. AGR2 was biotinylated and then loaded at 25 μg/ml in PBS buffer for 300 s prior to baseline equilibration for 60 s in SD buffer. Association of 18A4Hu^scFv^ in SD buffer at various concentrations in a twofold dilution series from 1000 to 62.5 nM was conducted for 300 s prior to dissociation for 300 s. The data were baseline subtracted prior to fitting using a 1:1 binding model and ForteBio data analysis software 10. Mean k_on_ and k_off_ values were determined with a global fit applied to all the data.

### Antibody Preparation

Antibodies were expressed in HEK293F cells. HEK293F cells were cultured in DMEM with 10% SFM and 1% penicillin and streptomycin at 37°C, 5% CO_2_, and 55% humidity. Plasmids were transfected into cells using polyethylenimine (PEI) (Polysciences, Warrington) until the cell density reached 10^6^ cells per mL. Three or four days after transfection, cell supernatants were collected. Subsequently, antibodies were isolated and purified using protein G beads (Smart Lifesciences, Shanghai, China) in a filtration column (ABclonal Technology, Wuhan, China). These antibodies were detected by SDS-PAGE and quantified by NanoDrop IgG mode (Thermo Fisher Scientific, Waltham, USA).

### Animals

BALB/c nude mice aged 6 weeks were purchased from Shanghai Lingchang Information Technology Co., Ltd., and housed in sterile cages under laminar airflow hoods with a 12-h light/dark cycle at 22°C~25°C, with ad libitum access to food and water. All experiments were evaluated and approved by our institution and performed under the Guidelines for Animal Care at Shanghai Jiao Tong University.

### *In Vivo* Treatment and Lung Metastasis Studies

To establish the lung metastasis tumor model, B16F10 cells (2 × 10^5^ cells per animal) were inoculated into BALB/c nude mice through tail vein injection in a 200-μl volume of medium. Then, mice were treated with the antibody 18A4Hu wild type and variants one for every 3 days for five times starting from day 2 by intraperitoneal (*i.p.*) injection (100 mg per kg in 200 μl 1× PBS). On the day of sacrifice, tumor metastasis in the lung was measured by quantified black dots on the lung surface and H&E staining.

### H&E Staining and Immunofluorescence Analysis

Mouse tissue samples were fixed in 4% formaldehyde, frozen, and processed into sections of 10-μm thickness by freezing microtome. Samples were subjected to H&E staining according to standard protocols. For immunofluorescent analyses, sections were permeabilized with 0.1% Triton X-100 in 1× PBS for 5 min and blocked in 1% BSA and 5% goat serum in 1× PBS for 30 min at room temperature. Primary antibodies (ABclonal Technology, Wuhan, China) against AGR2 and anti-human IgG were applied overnight at 4°C in blocking buffer (1% BSA in PBS and 0.05% Tween-20) and visualized using secondary antibodies in 1× PBS for 45 min at room temperature. Subsequently, nuclei were stained with DAPI at room temperature for 5 min and kept in the dark. The images were detected and captured by fluorescence light microscopy (Leica, Wetzlar, Germany).

## Data Availability Statement

The original contributions presented in the study are included in the article/**Supplementary Material**. Further inquiries can be directed to the corresponding authors.

## Ethics Statement

The animal study was reviewed and approved by the Institutional Animal Care and Use Committee of Shanghai Jiao Tong University.

## Author Contributions

S-CT and H-NZ developed the conceptual ideas and designed the project. H-NZ, J-BX, Z-LW, H-WJ, and SM performed the experiments. D-WL provided key materials. H-NZ and S-CT wrote the manuscript with suggestions from other authors. All authors contributed to the article and approved the submitted version.

## Funding

This work was partially supported by the National Natural Science Foundation of China (Nos. 31900112, 21907065, 31970130, 31670831, 31370813) and China Postdoctoral Science Foundation (Nos. BX201700155, 2018M642005). This work was partially supported by the National Key Research and Development Program of China Grant (No. 2016YFA0500600).

## Conflict of Interest

The authors declare that the research was conducted in the absence of any commercial or financial relationships that could be construed as a potential conflict of interest.

## Publisher’s Note

All claims expressed in this article are solely those of the authors and do not necessarily represent those of their affiliated organizations, or those of the publisher, the editors and the reviewers. Any product that may be evaluated in this article, or claim that may be made by its manufacturer, is not guaranteed or endorsed by the publisher.
