# Supplementary material for: EASINESS: E. coli Assisted Speedy affINity-maturation Evolution SyStem
Source: Front Immunol. 2021 Dec 3;12:747267. doi: 10.3389/fimmu.2021.747267 (PMC8677947; doi:10.3389/fimmu.2021.747267)
Supplement: Supplementary file 1 [file DataSheet_1.pdf]

Supplementary Materials for

EASINESS: *E. coli* Assisted Speedy affINity-maturation Evolution SyStem

Hai-nan Zhang[1,#], Jun-biao Xue[1,#], Ze-ling Wang[2,#], He-wei Jiang[1], Siva Bhararth Merugu[2,] Da-wei Li[2,*], and Sheng-ce Tao[1,*]

*** Correspondence:** taosc@sjtu.edu.cn (S-c. Tao)

Supplementary Figure 1 Construction and validation of two key components of EASINESS.

Supplementary Figure 2 Characteristic of 18A4Hu^{scFv} wild type and mutants.

Supplementary Figure 3 Characteristic of 18A4Hu wild type and mutants.

Supplementary Figure 4 Expression and purification of 18A4Hu wild type and mutants.

Supplementary Table 1 Strains and plasmids used in this study.

Supplementary Table 2 Primers used for several construction and sequencing in this study.

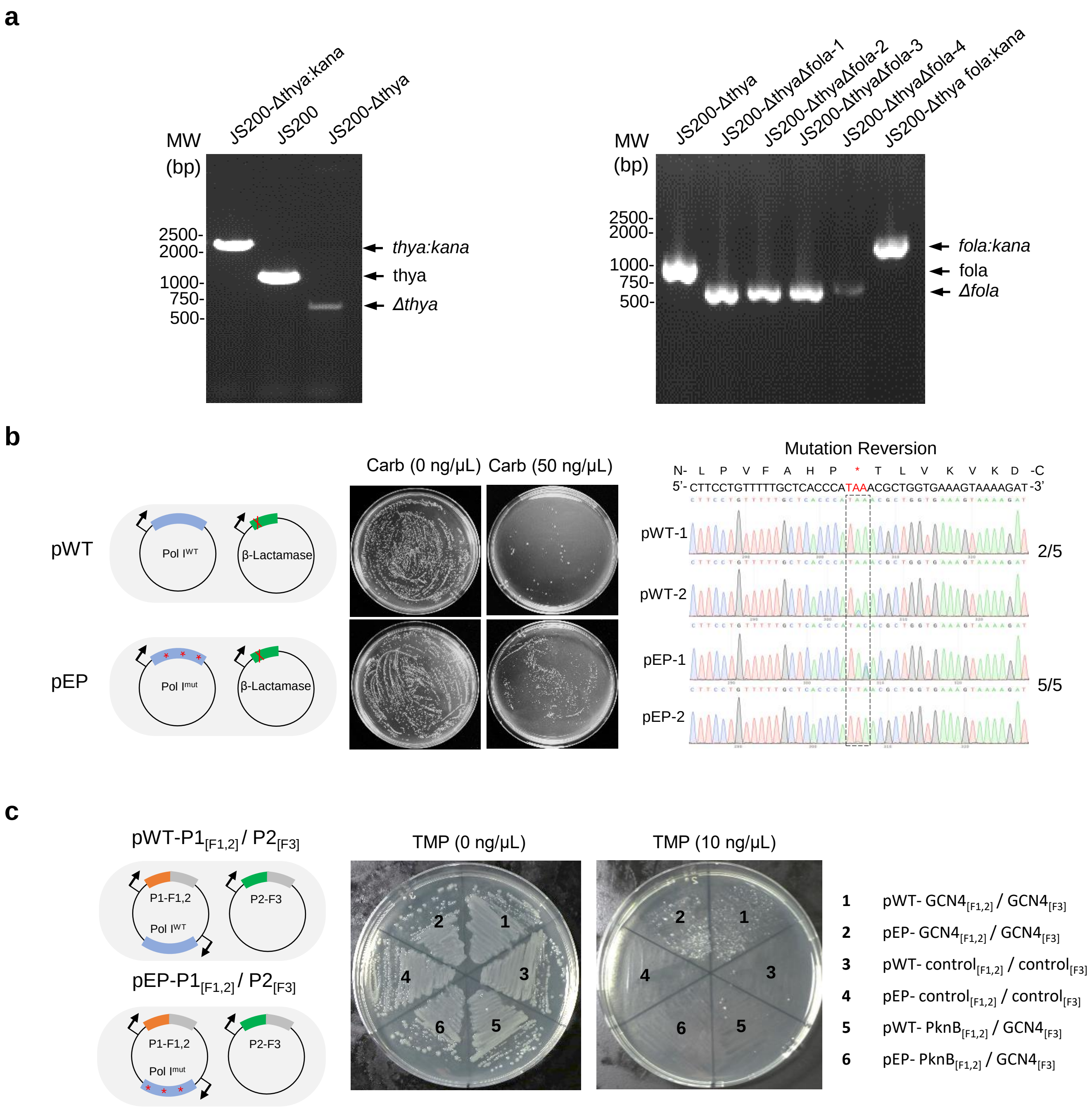

Figure S1. Construction and validation of key components in EASINESS. a. Gel analysis (1% agarose, stained with gelRed) of both thya and fola deletion in JS200-Δ*thya* and JS200-Δ*thya*Δ*fola* cells. **b.** PolI^{3mut} (pEP) achieves more efficient and preferential mutagenesis than PolI^{WT} in β-lactamase stop codon TAA reversion. JS200-Δ*thya*Δ*fola* cells carrying PolI^{3mut} or PolI^{WT} and the reporter plasmid pLA230 were mutated and screened in the presence or absence of 50 μg/mL carbenicillin. **c.** Validation of protein-protein interactions using mDHFR-fragment complementation assays in JS200-Δ*thya*Δ*fola* cells. $GCN4_{[F1,2]}$ / $GCN4_{[F3]}$, $control_{[F1,2]}$ / $control_{[F3]}$ or $PknB_{[F1,2]}$ / $GCN4_{[F3]}$ were co-transformed into JS200-Δ*thya*Δ*fola* cells with PolI^{3mut} or PolI^{WT} and grown on LB plates in presence or absence of 10 μg/mL trimethoprim (TMP).

a

The sequences of 18A4Hu^{scFv} variants

Clone	Mutants	L12R	L14R	S102G	E131K	D226N
18A4Hu^{scFv}-1	WT	CTC	CTG	AGC	GAA	GAC
18A4Hu^{scFv}-6	L12R	C**G**C				
18A4Hu^{scFv}-7	L12R, D226N	C**G**C				**A**AC
18A4Hu^{scFv}-8	L12R, E131K	C**G**C			**A**AA	
18A4Hu^{scFv}-9	L14R		C**G**G			
18A4Hu^{scFv}-12-1	L12R, S102G	C**G**C		**G**GC		

b

The correlation between the concentration of TMP and the binding affinity of 18A4Hu^{scFv} mutants

Clone	Mutants	Cycles	TMP (ng/μL)	K_D (nM)	Folds of improvement (Mutant-K_D/ WT-K_D)
18A4Hu^{scFv}-1	WT	1	0	207	-
18A4Hu^{scFv}-6	L12R	2	100	159	1.3
18A4Hu^{scFv}-7	L12R, D226N	3	500	32.5	6.4
18A4Hu^{scFv}-8	L12R, E131K	3	500	65.2	3.2
18A4Hu^{scFv}-9	L14R	3	500	67.3	3.1
18A4Hu^{scFv}-12-1	L12R, S102G	4	1000	16.3	12.7

Figure S2. Characteristic of 18A4Hu^{scFv} variants. a. Detailed DNA and protein sequences of the mutated sites of 18A4Hu^{scFv} variants. **b.** The correlation between [TMP] and the binding affinity of 18A4Hu^{scFv} variants to AGR2.

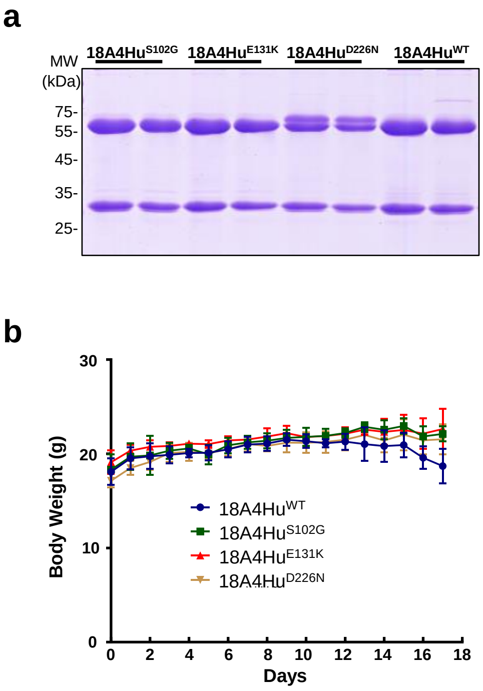

Figure S3. Characteristic of 18A4Hu wild type and mutants. a. The expression and purification of the wild type 18A4Hu and three mutants in mammalian cells. **b.** No significant change were measured in body weight of mice treated with $18A4Hu^{WT}$, $18A4Hu^{S102G}$, $18A4Hu^{E131K}$, or $18A4Hu^{D226N}$.

a

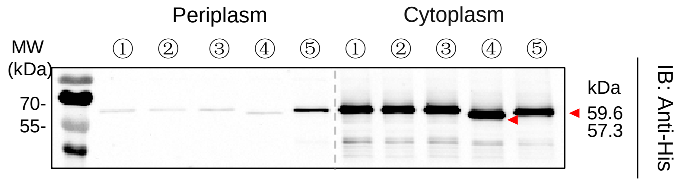

① SP-18A4Hu^{scFv}-WT ② SP-18A4Hu^{scFv}-L12R
③ SP-18A4Hu^{scFv}-L14R ④ 18A4Hu^{scFv}-WT (without the SP)
⑤ DsbA1-18A4Hu^{scFv}-WT

b

Sample	Periplasm	Cytoplasm (100x diluted)	Ratio (Periplasm /Cytoplasm)
①	204.14	6402.81	0.03%
②	121.02	5835.98	0.02%
③	191.44	6835.52	0.03%
④	188.73	7490.40	0.03%
⑤	2102.87	6539.15	0.32%

c

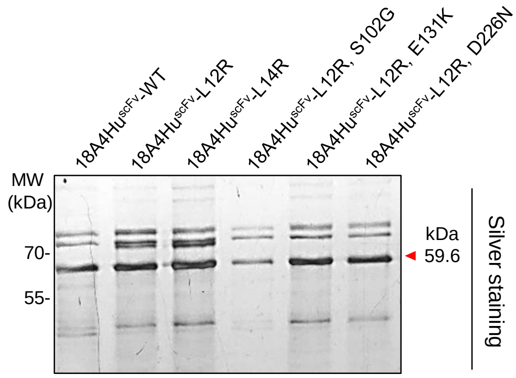

Figure S4. Expression and purification of 18A4Hu wild type and mutants. a. The expression of the 18A4Hu^{scFv} with/without signal peptide in periplasmic and cytoplasmic *E.coli* with western blot. For better comparison, the cytoplasmic portion was diluted for 100x before loading to the gel. **b.** Quantification of a. **c.** Purified 18A4Hu^{scFv}-WT and mutants (Silver staining).
